# Pulmonary fibrosis secondary to siderosis causing symptomatic respiratory disease: a case report

**DOI:** 10.1186/1752-1947-2-257

**Published:** 2008-08-05

**Authors:** Liam M McCormick, Martin Goddard, Ravi Mahadeva

**Affiliations:** 1 Department of Respiratory Medicine, Addenbrooke's NHS Trust, Hills Road, Cambridge CB2 2QQ, UK; 2 Department of Pathology, Papworth Hospital, Papworth Everard, Cambridge CB23 3RE, UK

## Abstract

**Introduction:**

Pulmonary siderosis secondary to the inhalation of iron compounds is a rare condition which, despite striking radiological and histopathological features, has not traditionally been associated with symptoms or functional impairment. Although not the first of its kind, we present an unusual case of pulmonary siderosis with symptomatic respiratory disease, most likely secondary to associated fibrosis.

**Case presentation:**

A 66-year-old Caucasian man was referred to the outpatient clinic with a 2-year history of exertional breathlessness. He had worked as an engineer for 20 years where he did a significant amount of welding but always wore a face shield. Clinical, radiological and histological features were consistent with a diagnosis of pulmonary siderosis, with associated fibrosis, most likely related to his occupational welding history.

**Conclusion:**

Our report illustrates that symptomatic respiratory disease due to mild peribronchiolar fibrosis can occur with pulmonary siderosis despite wearing a mask. Furthermore, it reinforces the need for all clinicians to compile a detailed occupational history in individuals presenting with breathlessness.

## Introduction

Pulmonary siderosis secondary to the inhalation of iron compounds is a rare condition which was first described in 1936 [1]. Despite striking radiological and histopathological features, it has traditionally been classified as a 'benign pneumoconiosis' [2] because of the absence of associated symptoms, functional impairment or pulmonary fibrosis [3]. Uncommonly, however, symptomatic disease with interstitial fibrosis has been described in arc welders [4]. We present an unusual case of pulmonary siderosis with symptomatic respiratory disease, most likely secondary to associated fibrosis.

## Case presentation

A 66-year-old Caucasian man was referred to the outpatient clinic with a 2-year history of exertional breathlessness. He had no other respiratory symptoms, had never smoked and was not aware of any previous asbestos exposure. He was not on any medication and had no allergies. He had worked as an engineer for 20 years where he did a significant amount of welding but always wore a face shield. A review of systems was unremarkable.

**Figure 1:**
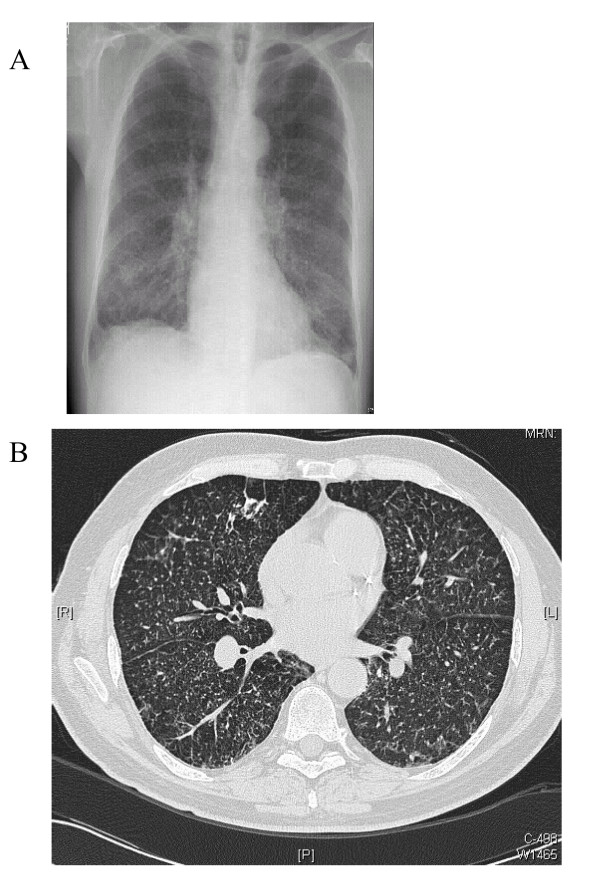
**Chest radiograph and computed tomography**. (a) Chest radiograph demonstrating diffuse generalised reticular nodular shadowing. (b) Chest computed tomography scan showing bilateral tiny nodular opacities throughout both lung fields predominantly in the mid and upper zones.

**Figure 2:**
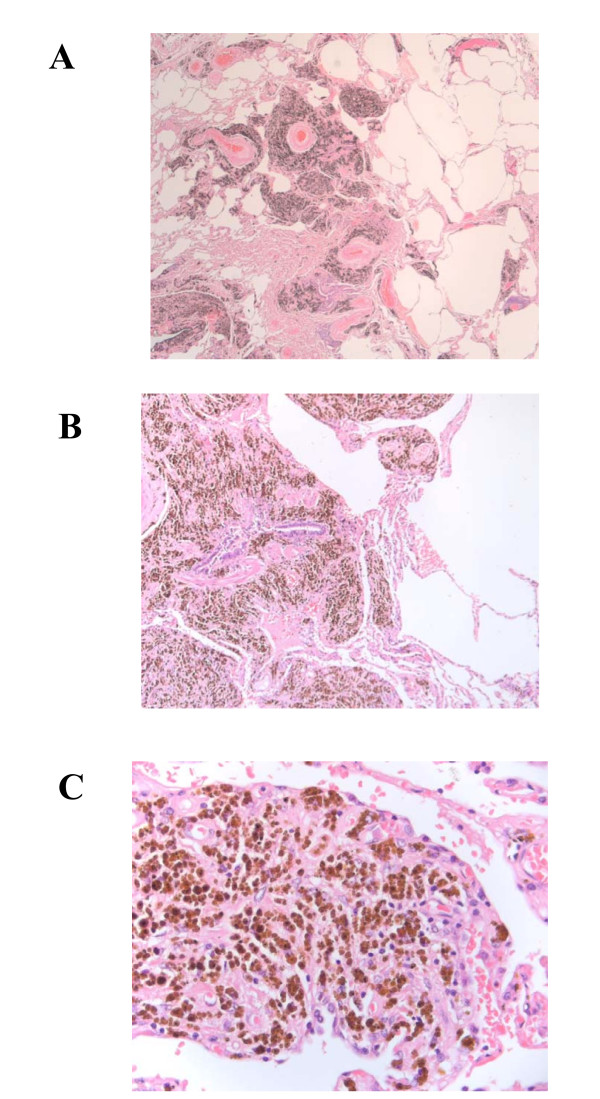
**Histological Analysis**. (A) Low-power (×100) micrograph showing pigment accumulation in an interstitial peribronchovascular distribution. (B) Higher-power (×200) view showing pigment in the interstitium around the airway; the alveolar air spaces are empty. (C) High-power (×400) view showing the 'golden granules' of haemosiderin.

**Figure 3:**
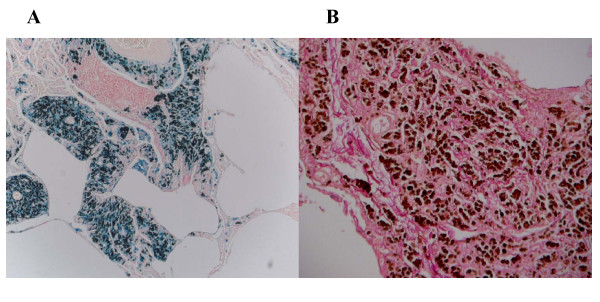
**Histology – Iron and Collagen Stains**. (A) Perl Prussian blue stain (×200) confirming deposition of iron. (B) High-power elastic van Gieson collagen stain (red) demonstrating significant fibrosis.

On examination, he was not clubbed or cyanosed, and his chest was clear to auscultation. Pulmonary function tests showed a moderately severe obstructive defect, gas trapping and a significantly reduced gas transfer factor: forced expiratory volume in 1 second (FEV1) 1.58 (49.1%); vital capacity (VC) maximum 3.0 (75.9%); FEV1/VC maximum 52.6%; total lung capacity (TLC) 7.2 (100%); residual volume (RV) 4.3 (157%); RV/TLC ratio 140.3%; carbon monoxide transfer factor 3.57 (44.0%); carbon monoxide transfer coefficient 0.77 (66.8%). His resting oxygen saturations were 95% on room air; however, he desaturated to 89% after 4 minutes of walking, which was associated with a peak modified Borg score (perceived breathlessness score) of three, indicating moderate breathlessness. A chest radiograph showed diffuse generalised reticular nodular shadowing with a suggestion of enlarged hila (Figure 1a). Computed tomography scanning revealed multiple small nodular opacities throughout both lungs, predominantly in the mid and upper zones (Figure 1b). Transbronchial biopsies were non-diagnostic, therefore video-assisted thoracoscopic lung biopsy was performed. Microscopic examination of these specimens showed marked deposition of coarse iron granules in a centrilobular distribution, with foci of associated fibrosis (Figures 2 and 3). The appearances were consistent with pulmonary siderosis most likely related to his occupational welding history. In 3 years of follow-up his lung function and chest radiograph have not progressed.

## Discussion

Inhalation of iron compounds occurs commonly in paint factories, during welding and steelmaking, and at various stages of iron mining and iron refining. Doig and McLaughlin first described 'welders' siderosis' in 1936 when they carried out a prospective study examining the clinical and chest radiological characteristics of 16 electric arc welders [1]. All but one of these original subjects were followed for 9 years: four of these demonstrated progressive radiographic reticular changes, nine showed no radiographic changes, and in two men (both of whom had spent significantly less time welding), there was evidence of at least partial resolution of the initial radiographic opacities [3]. All subjects, however, remained in good health, leading to the conclusion that siderosis (in its pure form) was not associated with respiratory symptoms or functional impairment. This view was supported by subsequent pathological investigations of the lungs of subjects occupationally exposed to iron oxide fumes, which did not demonstrate any evidence of pulmonary fibrosis [3]. As a result, the apparently inert nature of iron compounds led to the classification of pulmonary siderosis as a 'benign pneumoconiosis' [2].

Nevertheless, symptomatic disease with interstitial fibrosis has been described in arc welders [4]. Some authors have postulated that these rare cases are secondary to concomitant inhalation of silicates or asbestos that can occur in many occupations associated with exposure to iron [4]. However, Funahashi et al. challenged this view after investigating 10 symptomatic welders and performing energy dispersive X-ray analysis on lung tissue for elemental content [5]. Despite demonstrating restrictive defects in seven of their patients, and mild to moderate airway obstruction in a further two, they found no difference between the pulmonary silicon content of patients with symptomatic 'welders' pneumoconiosis' and that of age-matched control lungs [5]. Some degree of parenchymal fibrosis was present in all patients, and in 50%, this fibrosis was considered moderate to marked. As many of the iron-containing particles were seen in fibrotic alveolar septa, it was postulated that the fibrosis was a reaction to these particles rather than the co-existing silicosis [5].

## Conclusion

Our report illustrates that symptomatic respiratory disease due to mild peribronchiolar fibrosis can occur with pulmonary siderosis despite wearing a mask. Furthermore, it reinforces the need to compile a detailed occupational history in individuals with respiratory disease. It is particularly important to obtain an accurate diagnosis of interstitial shadowing on chest radiograph as pulmonary siderosis in our patient had a relatively good prognosis compared with other interstitial and small airway disorders.

## Abbreviations

FEV1: forced expiratory volume in 1 second; RV: residual volume; TLC: total lung capacity; VC: vital capacity.

## Competing interests

The authors declare that they have no competing interests.

## Authors' contributions

LM participated in the design and coordination of the case report and drafted the manuscript. MG acquired, analysed and reported on the histopathological slides. RM conceived of the case report, participated in its design and coordination, and revised it critically for important intellectual content. All authors read and approved the final manuscript.

## Consent

Written informed consent was obtained from the patient for publication of this case report and accompanying images. A copy of the written consent is available for review by the Editor-in-Chief of this journal.
